# eDNA Adoption: Weighing the Benefits and Challenges from Quebec Potential End-Users’ Perspective

**DOI:** 10.1007/s00267-025-02267-2

**Published:** 2025-08-28

**Authors:** Caroline Thivierge, Lynda Gagné, Limoilou-Amélie Renaud, Émilie Houde-Tremblay, Jérôme Dupras

**Affiliations:** 1 Department of Natural Sciences, Université du Québec en Outaouais, Gatineau, QC Canada; 2 Canada Research Chair in Ecological Economics, Montréal, QC Canada; 3 University of Victoria, Victoria, BC Canada; 4 School of Indigenous Studies, Université du Québec en Abitibi-Témiscamingue, Rouyn-Noranda, QC Canada

**Keywords:** Environmental DNA, Innovation adoption, Diffusion of innovation, Resistance to innovation, Stakeholder analysis, Technology acceptance

## Abstract

The collection of environmental DNA (eDNA) is a relatively new, non-invasive and effective method for detecting the presence of rare or endangered species, invasive alien species, and monitoring fish and wildlife populations, thus contributing to better conservation of natural environments. Academic researchers are its main users. The reasons for its slow diffusion among other potential users remain poorly documented to date. This study aimed to characterize the barriers and levers to the adoption of eDNA by distinct types of end-users, depending on the contexts in which they operate. We conducted semi-structured interviews with 33 participants to document and analyze their perceptions of eDNA. The Unified Theory of Acceptance and Use of Technology (UTAUT) inspired our analysis. Our findings revealed that potential end-users perceive the eDNA-based methods positively, although they are improvable. A lack of knowledge about its limitations and potential affects how useful eDNA is perceived to be and potential end-users’ confidence in its results. We propose action levers to increase potential end-users’ trust in the method and its compatibility with their current practices, and identify avenues to facilitate its diffusion.

## Introduction

Anthropogenic pressures on natural environments, such as habitat loss and fragmentation (Chase et al. 2020), exploitation of natural resources (Dulvy et al. 2021), climate change (Habibullah et al. 2022), pollution (Zhang et al. 2021) and the introduction of invasive alien species (Tamburello and Litt 2023), threaten biodiversity (IPBES 2019; Jaureguiberry et al. 2022). It is therefore essential to have effective monitoring tools adapted to these challenges. Environmental DNA (eDNA) was initially developed from the pioneering study by Handelsman et al. (1998), which argued that the low abundance of micro-organisms in the soil highlighted the need for a precise tool to confirm their presence. The use of eDNA, which involves sampling and analyzing genetic material in the environment (e.g., water, soil, air, sediments), represents a major advancement in biodiversity monitoring (Handelsman et al. 1998). Over the years, users have employed eDNA in biodiversity assessments that focus on micro- (Ladin et al. 2021) and macro-organisms (Tsuji et al. 2019), specific species (Baker et al. 2018), biological communities (Keck et al. 2022), faunal history from sediments (Lopez et al. 2024), and predator fecal sample eDNA analyses that identify the presence of prey in the environment (Nørgaard et al. 2021).

The primary advantage of eDNA is its sensitivity and its ability to detect low-abundance species that may go unnoticed with direct observation methods, such as rare or endangered species or new invasive alien species (Bohmann et al. 2014). Moreover, eDNA is particularly valuable in data-sparse settings, including remote and marine environments—such as the deep sea (McClenaghan et al. 2020)—where conventional biological monitoring is constrained by logistical, financial, or ecological limitations. Non-invasive, the collection of eDNA allows the detection of species without capturing specimens or using techniques that could disturb them in their environment (Thomsen et al. 2012). eDNA is proving useful for estimating the abundance of certain species, particularly in aquatic environments (Spear et al. 2021; Yates et al. 2021; Shelton et al. 2022). Although set-up and capital costs can be high, particularly for the purchase of specialized bioinformatics equipment and database development, eDNA can be more cost-effective than conventional methods over a long period and on a large spatial scale, by reducing the need for fieldwork (Sigsgaard et al. 2015; Beng and Corlett 2020) and expensive taxonomic expertise (Yu et al. 2012) to support decision-making related to conservation and ecosystem management efforts (Deiner et al. 2017). It is a versatile tool that can be applied to various ecosystems (aquatic, terrestrial, and aerial) and to a wide range of taxa (Beng and Corlett 2020). Beyond simple presence/absence, eDNA is suitable for multiple applications, such as assessing the environmental impact of anthropogenic activities (Rishan et al. 2023), mapping the biogeographic distribution of species, determining species richness, and studying interactions between species (Miya 2022).

While eDNA-based methods are continuously evolving and have many advantages, they also have limitations. Although studies show a positive correlation between eDNA concentration and biomass and abundance (Rourke et al. 2023; Hansen et al. 2018), current applications are largely species-specific and more reliable under laboratory conditions (Yates et al. 2019; Carvalho et al. 2022). Even if eDNA can be used to estimate the abundance of a species, further studies are needed to evaluate its effectiveness for that purpose (Beng and Corlett 2020). As it is an indirect observation method, eDNA does not provide data such as weight, size, life stage and health status from samples (Ruppert et al. 2019). This limits researchers’ ability to assess the impact of environmental disturbances or the effectiveness of certain conservation measures. So far, eDNA-based results are currently more reliable in aquatic environments than terrestrial ones (Rishan et al. 2023; Beng and Corlett 2020), and some taxa, such as small terrestrial mammals, are more difficult to detect by eDNA than by capture (Brochu et al. unpublished data). Operationally, sample contamination is a risk at each stage (collection, filtration, analysis), and incomplete reference databases can hinder species identification (Van Klink et al. 2022; Rishan et al. 2023). eDNA methods are continuously evolving, and international efforts are underway to standardize analysis protocols. Notably, initiatives such as the CSA standards in Canada (Helbing and Hobbs 2019; Langlois et al. 2021) and the DNAqua-Net project in the European Union (Leese et al. 2018) illustrate the growing institutional commitment to harmonizing practices and ensuring methodological reliability. Knowledge continues to advance regarding issues related to DNA degradation under different biotic and abiotic conditions (Caza-Allard et al. 2022), its persistence in an environment like seawater (McCartin et al. 2022), and the risks of false positives and false negatives resulting from the amplification process (Zhang et al. 2023).

Despite these advancements, eDNA-based methods’ adoption rate remains low among certain types of actors, and its use is mainly limited to academic scientific circles (Kelly et al. 2023). The uptake of eDNA across organizations can be perceived as slow and uneven, as evidenced by the continued emphasis in the literature on institutional, technical, and social barriers to its implementation more than a decade after its emergence (Beng and Corlett 2020; Bernos et al. 2023; Doi and Nakamura 2023). So far, the literature reveals three main adoption drivers: trust in the method, organizational capacity, and the perception of usefulness. Trust in the method relies on the standardization of protocols (Langlois et al. 2021; Doi and Nakamura 2023), the robustness of the data it generates, and the eDNA method’s official recognition by competent authorities (Bernos et al. 2023). Scientists and community users can develop trust through closer collaboration (Lodge 2024; Doi and Nakamura 2023). Organizations’ available resources, both human and financial, affect the eDNA method’s probability of adoption (Lee et al. 2023; Doi and Nakamura 2023). The adoption process, particularly in the public sector, also depends on internal expertise and the support of “champions“ who function as innovation diffusion agents (Lee et al. 2023). Champions’ communication efforts are important as they impact people’s perception of eDNA and its potential use (Stein et al. 2023). Finally, adoption requires that potential end-users perceive eDNA as useful. For example, potential end-users anticipate difficulties in obtaining quantitative results with eDNA, which could be a barrier to adoption (Mont’Alverne Bretz Giovanini 2022). Levers for eDNA adoption could include demonstrating applicability across contexts and improving reference databases (Mont’Alverne Bretz Giovanini 2022). Pilot implementation in controlled settings, such as sandbox approaches, may offer a structured means to evaluate eDNA’s suitability through evidence-based and transparent processes (Lee et al. 2023).

The few existing studies on the adoption of eDNA have primarily focused on technical aspects, overlooking the analysis of potential users' decision-making processes. This study addresses that critical gap by examining end-user perceptions to better understand the factors influencing adoption. Our research aimed to understand how current and potential end-users perceive the attributes of eDNA-based methods, such as their usefulness, ease of use, efficiency, and reliability, and how well these methods align with their practical needs. Specifically, we sought to identify (1) the perceived advantages and barriers that may influence adoption-related decision-making, and (2) the levers and conditions that could facilitate the broader uptake of eDNA by potential end-users. To this end, we conducted a case study in Quebec, interviewing 33 potential and current end-users from diverse types of organizations engaged in environmental monitoring and management.

## Materials and Methods

### Conceptual Framework

As an emerging method in biodiversity monitoring, eDNA qualifies as an organizational innovation. While its adoption shares common features with that of information technologies (IT)—such as the role of organizational decision-making and the importance of knowledge transfer—it follows distinct dynamics. IT adoption is typically driven by top-down strategies aimed at improving operational efficiency, whereas innovations like eDNA often emerge through bottom-up processes led by internal actors who champion change (Rogers 2003). Given these characteristics, the adoption of eDNA is best examined through the lens of innovation and diffusion theories. Rogers' (2003) theory of the diffusion of innovations is based on five main determinants: the relative advantages of the innovation, its compatibility with user needs, ease of use, trialability, and observability. In all cases, adoption assumes that the user knows the innovation and its characteristics but is also able to assume the risks associated with changing habits (Rogers 2003). Risks can relate to an investment (money or time) or to results, which can be unexpected compared with those obtained with conventional methods. Faced with change, individuals tend to favor maintaining their habits (Ram 1987). The adoption of an innovation may also require repeated exposure to a favorable message regarding the innovation from various sources over a prolonged period (Iacopini et al. 2019). Exposure to an innovation occurs through diffusion agents whose skills, knowledge, and social networking favor message transmission (Rogers 2003). For a person to function as a diffusion agent for an innovation within their network, they must feel motivated, legitimate, and have a sense of self-efficacy regarding its use (Rogers 2003; Jones and Niemiec 2020).

Predicting the adoption and diffusion of an innovation has been the subject of much research. The frequently utilized theory of reasoned action posits that individual and normative beliefs influence attitudes and intentions, which in turn determine behavior (Fishbein and Ajzen 1975). Inspired by this theory, the technology acceptance model identifies the perception of usefulness and ease of use as effective determinants of intention (Davis 1989). However, this model could be difficult to transfer from a sociocultural context to another as demonstrated by Straub et al. (1997). Their study did not yield consistent results across contexts, with findings in Japan differing from those observed in the U.S. and Switzerland (Straub et al. 1997).

To address this limitation, Venkatesh and Davis (2003) proposed the Unified Theory of Acceptance and Use of Technology (UTAUT), integrating elements from eight different theories and models, including the theory of reasoned action, the technology acceptance model, and the diffusion of innovation theory. UTAUT has been used in the context of information system adoption, such as e-learning (Abbad 2021), social media adoption (Puriwat and Tripopsakul 2021), and more recently artificial intelligence (Venkatesh 2022). UTAUT integrates constructs such as performance expectancy, effort expectancy, social influence, and facilitating conditions (Venkatesh and Davis 2003). UTAUT has demonstrated superior predictive ability and is widely used in technology adoption research (Venkatesh et al. 2012). Mainly mobilized to study the *individual* adoption of information technologies (IT) (Gupta et al. 2008; Pynoo et al. 2011; Kim and Bae 2020), UTAUT is also relevant to the analysis of eDNA, as its core constructs reveal striking similarities—such as the pursuit of efficiency, the complexity of adoption requiring learning, the influence of institutional leadership or peers, and the reliance on material and logistical resources. In this study, we used the constructs and variables of the UTAUT (presented in Table 1) as a theoretical basis to structure the interview questionnaire and for qualitative data analysis.

**Table 1:** Definitions of constructs from the Unified Theory of Acceptance and Use of Technology (UTAUT) (Adapted from Venkatesh and Davis 2003)

Construct	Definition	Variables
Performance expectancy	Degree to which an individual believes that using an innovation will help them to attain gains in job performance.	Perceived usefulness Relative advantage Outcome expectation Job fit Extrinsic motivation
Effort expectancy	Degree of ease associated with the use of the innovation.	Perceived ease of use Complexity Ease of use
Social influence	Degree of importance the individual attaches to others' perception of their use or non-use of the innovation.	Subjective norms Social factors Image
Facilitating conditions	Degree to which an individual believes that an organizational and technical infrastructure exists to support use of the innovation.	Perceived behavioral control Facilitating conditions Compatibility

### Data Collection Method

We conducted semi-structured interviews with 33 potential and current eDNA end-users in the province of Quebec, Canada. The principal author conducted all interviews between May 15, 2023, and April 30, 2024. Interviews lasted between 45 and 90 min. Potential end-users are organizations that already carry out environmental data collection activities as part of their mandate, but do not use eDNA and have limited knowledge about this method. Current end-users are either familiar with eDNA or very knowledgeable of its potential applications. Semi-structured interviews allow for detailed exploration of the meaning individuals attribute to their experiences while providing a structure to organize their thoughts (Galletta 2013; Bourgeois 2021). This method allows for the emergence of topics while ensuring coverage of important themes (Smith et al. 1995; Gaudet and Robert 2018).

Table 1 presents definitions of the key constructs from the UTAUT, as adapted from Venkatesh and Davis (2003). The model was used to guide the development of a semi-structured interview questionnaire aimed at exploring factors that influence the adoption of eDNA-based methods among current and potential end-users in Quebec, Canada. The four core constructs are described as they informed the design and interpretation of interview questions.

The semi-structured interview guide comprised three sections: (1) organizational practices, needs, habits and challenges related to biodiversity data acquisition; (2) awareness of environmental issues and their perceived impact on data-related activities; and (3) perceptions of eDNA methods, based on end-users’ own knowledge. Questions in the third section were built according to the main constructs of the UTAUT (Table 1). Participants were first asked about their familiarity with eDNA. For those with limited knowledge, a standardized definition was provided to ensure a shared understanding: “The sampling and analysis of genetic material present in environmental substrates—such as water, air, soil, or sediment—to detect the presence of one or more species”. This was followed by specific questions on issues such as its perceived usefulness in their work, the perceived complexity of eDNA sample collection or analysis, and their trust in eDNA for detecting rare species or invasive alien species. Participants were then asked to reflect on their organization’s capacity to integrate innovation, the processes by which new approaches are introduced, the factors that might encourage their adoption, and the perceived advantages and disadvantages of eDNA.

### Sampling and Participant Recruitment

Potential end-users invited included employees of government agencies (scientists and managers), regional administrations, environmental non-governmental organizations (NGOs), academic institutions (scientists only), and private environmental consulting firms. The sample targeted organizational leaders as well as those responsible for data acquisition activities in the field, i.e. individuals in decision-making positions or with the power to recommend the adoption of eDNA within their organization. Of the 70 individuals invited by email to participate in the study, 33 (47%) agreed. Most participants were biologists, but the sample also include a forestry engineer, three individuals trained in environmental studies and two agronomists (Table 2). A heterogeneous sample was favored to represent a wider range of opinions, which increases the robustness and generalizability of the results (Prévost et al. 2015). Recruitment among potential end-users was challenging because of their lack of knowledge about eDNA, leading many invitees to refer us to colleagues who already used the method. The sample thus consisted of 26 potential and 7 current eDNA end-users. Recruitment ended when data saturation was observed, as recommended by Marshall et al. (2013).

**Table 2:** Distribution of participants by type of organization

Participants organizations	*N*	%
Governmental agency (GOV-M and SCI-GOV)	11	33.33%
Regional administration (RA)	3	9.09%
Environmental NGO (NGO)	8	24.24%
Academic institution (A-SCI)	4	12.12%
Private consulting firm (PRI)	7	21.21%
Total	33	100%

### Interview and Analytical Methods

Interviews were conducted in French using Microsoft Teams and fully transcribed. Videos and recordings were deleted, and verbatim transcripts were coded using MaxQDA software (v.24.8.0). A three-step coding process was carried out. As described by Saldaña (2021), coding is an iterative process of assigning labels or short phrases to data segments, allowing them to be categorized and for common themes to emerge. The first phase consisted of classifying the data according to the UTAUT constructs and variables. The second (inductive) phase consisted of identifying new codes from the classified data. The final phase consisted of refining the main and emerging categories and classifying the codes as positive, neutral, or negative perceptions.

## Results

The results are presented according to the UTAUT constructs: (1) performance expectancy, (2) effort expectancy, (3) social influence, and (4) facilitating conditions.

### Performance Expectancy

Participants frequently mentioned the sensitivity of the eDNA-based method as an advantage. Many believed that “*there is a higher chance of detecting the species if it is present*” with eDNA (NGO-02). This sensitivity can save data collection time, especially when the objective is to detect the presence of low-density taxa. This is perceived as an important advantage, particularly for the early detection of invasive alien species, as this participant indicated: “*Well, when I'm looking for invasive species, environmental DNA is my best tool available*.” (SCI-06). For others, eDNA-based methods facilitate the detection of endangered and vulnerable species whose inventory period is short and dependent on weather conditions. The case of the boreal chorus frog (*Pseudacris maculata)*, which is conventionally detected by call surveys was provided as an example during interviews. For a participant, detecting the DNA of this species was “*factual, scientific proof that the species used the environment. It is much more robust than being there every spring during the right four days when it sang to know if it was there*.” (GOV-M-01).

Trust in eDNA’s ability to identify endangered species or conduct inventories through metabarcoding varied among participants. While many perceived the results obtained by eDNA-based methods as reliable and providing hard-to-contest factual evidence, others had more nuanced opinions. Our results suggest that the level of confidence is higher for the targeted real-time or quantitative real-time polymerase chain reaction (qPCR) technique than for metabarcoding. This nuance was more prevalent among scientists (GOV and A) who seemed more aware of the current reference database limitations and the precautions to take to avoid sample contamination and obtain accurate and reliable results. Also, the level of confidence in the results obtained by eDNA was higher for aquatic environments, as some participants expressed doubts about the robustness of protocols in terrestrial environments, as this participant, for example, expressed: “*you collect surface soil, it tells you one thing, but you collect five centimeters deeper, it tells you another thing*” (A-SCI-01). Comparing direct observation methods with eDNA, some participants also questioned the impact of species movement on the results obtained and their interpretation.

*The only thing is, let me give you an example: a seabird that puts its feet in the ocean, gets lots of particles stuck to its feathers and feet, and then goes to rest on a lake. And then you measure, and by chance or misfortune, you discover that there are cod and octopuses in the lake because those particles end up in the lake. Where is the truth in all this? (A-SCI-03)*

Researchers from organizations such as environmental NGOs, academic institutions and governmental agencies regularly need data on the relative abundance of species, their biomass, their health status, and their role in ecosystems. Some participants feared that this important information is inaccessible through eDNA. Participants also expressed doubts about the effectiveness of eDNA-based methods in identifying subspecies within the same taxonomic rank. Many cited the importance of using biologists in the field to accurately identify both plant and animal species. Because eDNA methods do not provide answers to all the questions of interest, most participants perceived it as complementary to conventional methods. For example, biologists in regional administrations who collect data at the landscape scale to document water run-off or soil erosion issues (RA-01) find the method interesting but much too specific for their needs.

One participant stated that data acquired by eDNA-based methods are not better, but different from the data one would obtain from a biologist in the field (A-SCI-02). Most participants indicated that eDNA would not replace conventional methods but would add to their toolbox. Participants also indicated that identifying what is difficult to see with the naked eye or being able to conduct an inventory in a larger territory are other advantages from using eDNA (NGO-06).

Participants perceived eDNA methods not only as complementary but also as going beyond parallel use by enriching more conventional methods. It was perceived as a decision-making tool to guide conventional sampling campaigns, as explained by this participant who stated that eDNA may be used to do a reconnaissance of the presence of a species and to “*maybe try to see where they are located, what the abundance is, and characterize further. So, it could maybe save time in targeting the areas to work on*.” (NGO-04)

Overall, participants expressed that the method still seems improvable, considering it at a developmental stage. Given that eDNA technology is evolving, many expressed that it is better to wait until the method is perfected before integrating it into their practice. This perception was shared by all types of actors, regardless of their knowledge level about the limitations and possibilities of eDNA.

### Effort Expectancy

Participants believed it is faster to obtain results with eDNA-based methods. The possibility of conducting inventories more quickly was also identified as a strength. A participant indicated that to obtain a similar result with conventional techniques, the effort required would “*be enormous”* (RA-02). Nevertheless, implementing eDNA methods was perceived as requiring significant resources in terms of qualified personnel, specialized equipment, and funding because of its complex nature. Participants also expressed doubts about their organization’s ability to integrate this innovation. The recruitment of qualified employees was identified as a challenge. Participants from environmental NGOs also mentioned having difficulty carrying out data acquisition activities due to lack of time, money, and available workforce, and were already overloaded with numerous inventory activities taking place simultaneously. Participants indicated that decision-making processes within large organizations can be long and complex, slowing the adoption of new methods. Moreover, implementing eDNA-based methods involves complex logistics, particularly in terms of sample transport and access to equipped laboratories. Incidentally, some environmental NGOs and private sector participants identified consistency and reliability issues with transport services in their territory: water samples collection for water quality monitoring sometimes needs to be redone because the delivery service does not meet the deadlines required for analysis. Some participants questioned the impact this could have on the reliability of eDNA results.

Participants identified the issue of costs and organizational financial capacities as limitations to using eDNA-based methods. One participant considered it the main barrier to using eDNA: “*What would prevent us is really the financial aspect. That would be the number one reason. I think I don't see any other. I think that’s really what would limit us*.” (NGO-07). Participants perceived that eDNA-based methods are expensive. Although they couldn't quantify the cost, this perception was mainly linked to the price of sequencers and the specialized labor required for analysis. It was also difficult for participants to gauge the cost-effectiveness of eDNA methods compared to conventional methods. The latter appeared more cost-effective to some participants, given the large amount of qualitative data that can be collected by field teams. From an institutional perspective, several participants also mentioned experiencing budgetary challenges in ensuring the sustainability of jobs or financing field data collection activities. This challenge was identified by both public and private sector actors. Social factors may influence the interest of both governmental and private sector stakeholders in the adoption of eDNA-based methods. While government actors were more concerned about the burden on taxpayers and their budgets, private consultants were more concerned about competitiveness, namely ensuring that costs are acceptable to their clients.

### Social Influence

Participants' needs for environmental data were influenced by their organizations mandate, but also by external factors such as the regulatory framework and social influence, i.e., the needs expressed by clients or available funding opportunities. This was the case for all types of actors interviewed. For example, a scientist indicated increasingly avoiding working with endangered and vulnerable species due to the administrative burden associated with permit applications: “*every year they bring us a new constraint to collect data on animals*” (A-SCI-04). Likewise, private consultants acquire environmental data to meet their clients' needs. One client need mentioned by all the private consultants was the delineation of the presence of a wetland to comply with the Environmental Quality Act (*Loi sur la qualité de l’environnement*). While private consultants expressed an interest in eDNA-based methods, they considered justifying its use to their clients as a challenge, as it is not a government requirement. Participants from environmental NGOs stated that data collection activities are poorly funded by funders, forcing them to make choices when it comes to fulfilling their mission:

*“You know, with the budgets you have, you try to do as much as possible. It’s a lot of observation, and yes, there is some place for intuition. You often get a good overview of the habitat, and you say to yourself, There surely is wild garlic, but I won’t find any, because it’s too rocky.'” (NGO-01)*

Many participants first heard of eDNA-based methods through the national media or at scientific conferences. When questioned about integrating innovations within their organization, participants stated that an innovation is most often championed by an employee who heard about it and developed an interest in it. They then act as a diffusion agent within the organization by encouraging their management to try it. However, this role is sometimes perceived as arduous and time-consuming, and not everyone wants to be responsible for it:

*“I am always receptive to things like that as long as it's not me who is stuck bringing it to the regional administration, and take care of it, and be the father of this thing.” (RA-01)*

Finally, participants from environmental NGOs and private consulting firms believe that using eDNA would increase their credibility with the government and could help eliminate potential doubts about the quality of their data. Using eDNA, when it isn’t mandated by the government, would also contribute to projecting a positive public image of their organization. Most private consultants mentioned that if a competitor used eDNA methods, it would encourage them to use it: “*If they (directors of the organization) see that competitors are using it or if ministries require it, these are all things that could encourage us to learn to use it as well*.” (PRI-05). Peer influence can also affect regional administration: “*[when] someone else has had a good idea… we imitate it.”* (RA-02). Social influence seems less important for scientists and government managers as their interest “*depends on the issue*” they are studying (GOV-M-07).

### Facilitating Conditions

Participants stated that clear and standardized sampling protocols, as well as transparent and accessible guidelines, would facilitate eDNA-based methods’ integration into existing practices. Participants said they need to be convinced of the validity and reliability of the data acquired by eDNA. They mentioned that the possibility of trying it and being able to compare the eDNA results with their field observations could help increase their level of confidence about the results. Additionally, knowledge dissemination, particularly through training and knowledge transfer activities, would strengthen potential end-users' trust in the tool and their own ability to use it.

Institutional recognition could encourage many participants to use it by increasing trust in eDNA:

*“Well, I think so. If they (the government) recognize it, that it is reliable and approved, I think it would carry weight in our analyses. I think there would be an interest in working with it.” (NGO-04)*

Institutional recognition includes government recognition through the protocols they recommend, recognition of the method’s usefulness in participants’ organizations, and donor funding for eDNA data acquisition. Changes in regulatory and legislative requirements could also facilitate its use, especially if it is cost-effective.

The issue of costs was central, both for private consultants and environmental NGOs, and was associated with the accessibility of the method. Most participants emphasized the importance of eDNA’s cost-effectiveness for it to be adopted; however, their current practices appeared more cost-effective to them. The lack of knowledge about costs, both for qPCR and metabarcoding, resulted in a perception that the eDNA method is expensive, an opinion shared by most participants.

## Discussion and Conclusion

This study investigated the potential for adopting eDNA-based methods in Quebec, Canada, using the Unified Theory of Acceptance and Use of Technology framework to assess key drivers: performance expectancy, effort expectancy, social influence, and facilitating conditions. Participants reported generally positive perceptions of eDNA-based methods, highlighting its non-invasive nature, efficiency, and sensitivity for detecting rare or invasive species. Although some participants reported limited familiarity with eDNA methods—a constraint that reflects both a limitation of the study and a broader knowledge gap—these perceptions nonetheless provide valuable insights into how innovations circulate, how organizational contexts shape adoption dynamics, and how such gaps influence attitudes and intentions toward eDNA use. Institutional constraints, perceived costs, and limited trust in the reliability of eDNA methods were also identified as barriers to broader uptake. Obstacles to the adoption of eDNA rest on perceptions, influenced by the level of knowledge about the method and its possibilities, and institutional and organizational issues that circumscribe biodiversity data acquisition actions in Québec. Importantly, these barriers also reveal concrete levers for action to support the integration of eDNA into existing biodiversity monitoring systems.

### Demonstrating Usefulness and Compatibility

Our results suggest that eDNA-based methods were neither perceived as useful by all participants nor in all circumstances. To a certain extent, eDNA-based methods were seen as complementary tools that can be used as decision aids, not as a standalone method that would replace conventional approaches. Participants’ perception of eDNA’s limited usefulness can be explained by insufficient knowledge about eDNA and its possibilities, giving them the impression that the method is incompatible with some of their needs. While eDNA was seen as useful for decision-making, no participants referred to its potential for species discovery via metabarcoding. Instead, those familiar with the method emphasized current database limitations, indicating that the approach still requires refinement.

The perception of usefulness and compatibility with needs are two determinants influencing the adoption of innovations (Venkatesh and Davis 2003). Research participants perceived eDNA methods as a way to validate the presence or absence of a species in an environment. This could explain the perception that eDNA is a useful method when very little data is available or to serve as a decision support in planning more exhaustive inventories by field specialists. Additionally, participants perceived that eDNA cannot be used to obtain data such as the relative abundance or biomass of a species. However, recent literature tends to show that relative abundance can be measured with eDNA, particularly in aquatic environments (Sepulveda et al. 2021). While molecular data can provide reliable abundance estimates for certain species, participants underscored the continued relevance of direct observation, which remains essential for assessing species health and capturing ecological dynamics that eDNA alone may not reveal. Regarding biomass, opinions were divided. The method has proven effective in determining the biomass of walleye (Spear et al. 2021) but inconclusive for Murray cod (Rourke et al. 2023). Wider diffusion of this innovation requires recognition of the current barriers to biodiversity data acquisition, as well as consideration of the needs and motivations of different types of organization.

This study highlights the importance of clearly communicating the strengths, limitations, and potential applications of eDNA, as these factors shape how useful the method is perceived to be—influencing both the intention to use it and the level of trust, which can be undermined by misuse or negative experiences. In that perspective, providing a decision-making tool as proposed by Stein et al. (2023), could also support adoption by organizations. A decision tree, for example, could assist potential users in systematically assessing the relevance of eDNA-based methods to their specific context by clarifying the conditions under which these methods are appropriate and highlighting their limitations to answer their questions. When co-constructed, a tool like this allows researchers to better understand the needs of environmental governance stakeholders and those to understand the issues related to the development of the eDNA method and its applicability in their activities.

Compatibility with needs was among the priorities identified by participants, and these needs are shaped by the existing institutional and regulatory framework, which influences not only the motivations for data acquisition (why), but also the type of data collected (what), as well as the procedures and timing for doing so (how and when). The Québec regulatory framework exerts a social influence on how environmental data are acquired. This represents a barrier to eDNA adoption for participants from private consulting firms as species-level or community-level inventories are not always required to obtain authorization certificates for their clients. In fact, to fulfill one of their principal mandates, which is delineating wetlands on a site targeted by a development project, the legislation does not require information on the species for which it is a habitat. To meet regulatory requirements, they primarily focus on wetland indicators, as mandated by the Environmental Quality Act. The use of eDNA remains difficult to justify to clients when it is not formally required by law. eDNA has little use in this context, where wetland indicators consist of vegetation, soil, and hydrology (Lachance 2021). Additionally, the lack of funding opportunities for species-level data acquisition leads conservation and environmental organizations to abandon species monitoring in favor of data acquisition at other spatial scales, like the critical habitat of umbrella species whose protection can also benefits other species, without needing to determine their presence (Simberloff 1998). These institutional and regulatory limitations restrict the adoption of new methods. They represent an economic risk, judged difficult to bear by these organizations. This can lead to the rejection of innovation, as individuals tend to maintain their habits if they do not have the means to face the risk associated with change (Rogers 2003). The legislative framework and organizational constraints, whether human or financial, constitute obstacles to eDNA adoption (Lee et al. 2023; Doi and Nakamura 2023) and to environmental data acquisition activities in general.

While the literature is replete with claims (Veldhoen et al. 2016) and provides evidence that eDNA methods are often far more cost-effective than conventional methods (Biggs et al. 2014; Bálint et al. 2018), this fact was lost on most participants, who did not recognize the trade-offs between eDNA methods low collection costs (for example, water sampling) vs. those of conventional methods (for example, fish netting and electrofishing) and of sequencing costs vs. those of taxonomic identification expertise. Cost-effectiveness analysis is a very specific methodology that compares the costs of a method vs. one or more other methods, per unit of benefit, assuming that this benefit is the same for all compared methods and that no other significant benefit exists for any of the methods (Cellini and Kee 2015). In the eDNA vs. conventional methods cost-effectiveness literature, this common benefit is the probability of detecting the species (Smart et al. 2016; Evans et al. 2017). However, conventional methods do offer other benefits that eDNA does not provide, such as information about the health of the individuals sampled, while eDNA is non-invasive and thus more respectful of the species (Helbing and Hobbs 2019). Study participants for whom species detection is an important need may not have considered or wished to consider that adopting eDNA could be less costly, but that realizing the savings would require making difficult decisions.

### Building Trust and Support

The results of this study point to action avenues to reach new end-users, relying on elements that already elicit a high level of trust and perceived usefulness. Participants highlighted the usefulness of eDNA as a decision-making tool and had more confidence in the qPCR method than in metabarcoding. The targeted method (qPCR) is simpler to access and more intuitive in results interpretation (detection or not of a targeted species), which could facilitate its diffusion by reducing the perceived effort to try it (Langlois et al. 2021). Standardized protocols and official recognition of eDNA-based methods by competent authorities are conditions that facilitate adoption by increasing the trust of potential end-users (Langlois et al. 2021; Bernos et al. 2023; Doi and Nakamura 2023). Establishing standardized protocols, as the one already existing for the qPCR method (CSA W219 :23), can facilitate the acceptance of the method by end-users and thus contribute to its broader use (Helbing and Hobbs 2019). Such protocols improve the quality and reproducibility of data and reduce false positives and negatives (Langlois et al. 2021). The qPCR method also fosters trust among users by allowing direct comparisons with conventional observation methods, as desired by some participants. It offers a promising entry point to spur the adoption of eDNA. Integrating pilot implementations in controlled environments, such as sandbox approaches (Lee et al. 2023), alongside collaborative trial opportunities with research teams, may strengthen trust in the method by enhancing its observability and trialability—two key determinants of innovation adoption and diffusion (Rogers 2003). These experiences can also improve perceived behavioral control, a critical facilitating condition for adoption (Venkatesh and Davis 2003), through transparent and evidence-based evaluation processes (Lee et al. 2023; Doi and Nakamura 2023; Lodge 2024; Ralson et al. 2025).

eDNA adoption could also be facilitated by adapting genomic laboratory service offerings to specific end-users’ needs. Targeted analyses on key species, such as indicator species or emerging invasive exotic species, could constitute a relevant entry point, particularly for environmental NGOs. Currently, many of them adapt their data collection to their budget capacity or turn to other data collection scales (e.g., habitat characterization) to infer the presence of a species. In this context, data acquired by eDNA could help them demonstrate the legitimacy of a project to funders by proving the presence of a species in a targeted environment. That said, to meet current end-user needs, it will also be necessary to continue efforts to improve eDNA infrastructure, and in particular, to expand reference databases. This prerequisite was also expressed by participants from other Canadian provinces (Mont’Alverne Bretz Giovanini 2022).

While adoption decisions for methods such as eDNA are typically made at the organizational level, their effective integration into routine practice depends on individual actors—often those who both influence internal decision-making and act as catalysts for innovation. This was reflected in our findings, as participants frequently described the adoption of new methods within their organizations as being driven by individual actors who served as internal champions. Institutional recognition of eDNA also emerged as a key enabling condition, reinforcing both the influence and effectiveness of these individuals in promoting its uptake. These observations aligns with the work of Rogers (2003) and Lee et al. (2023), who consider diffusion agents a key element of diffusion. To be effective, these diffusion agents must be motivated, recognized as legitimate by their peers, and feel competent regarding the innovation (Jones and Niemiec 2020). As Iacopini et al. (2019) suggest, repeated exposure to information from multiple sources is often necessary for adoption; a single encounter—such as at a conference or through a colleague—is unlikely to suffice. To fulfill their diffusion role, diffusion agents must be able to demonstrate the usefulness of the method, its cost-effectiveness, and the range of species that it can identify, and articulate an implementation plan. Broader diffusion of eDNA will require sustained knowledge transfer to potential end-users, as well as targeted training for individuals who show interest in the method and are willing to serve as diffusion agents within their organizations. Diffusion agents need training to fulfill this role but also support to help their organization adopt this innovation.

Finally, to better understand the influence of each individual and contextual factor and to predict its adoption, it would be relevant to conduct in-depth quantitative studies based on the UTAUT. These would allow measuring the weight of reasons for and against and evaluating their impact on decision-making according to the type of end-users. That said, while eDNA has the potential to enhance biodiversity monitoring, its broader adoption does not necessarily guarantee better outcomes; in the context of accelerating biodiversity loss, a critical examination of institutional frameworks and environmental data governance is needed to understand the conditions under which eDNA can contribute effectively to biodiversity conservation policies.

## Data Availability

No datasets were generated or analysed during the current study.
